# Regulating the Solvation Structure of Li^+^ Enables Chemical Prelithiation of Silicon-Based Anodes Toward High-Energy Lithium-Ion Batteries

**DOI:** 10.1007/s40820-023-01068-8

**Published:** 2023-04-18

**Authors:** Wenjie He, Hai Xu, Zhijie Chen, Jiang Long, Jing Zhang, Jiangmin Jiang, Hui Dou, Xiaogang Zhang

**Affiliations:** 1 Jiangsu Key Laboratory of Electrochemical Energy Storage Technologies, College of Material Science and Technology, Nanjing University of Aeronautics and Astronautics, Nanjing, 210016 People’s Republic of China; 2 School of Materials Science and Engineering, Henan Polytechnic University, Jiaozuo, 454003 People’s Republic of China; 3 Jiangsu Province Engineering Laboratory of High Efficient Energy Storage Technology and Equipments, School of Materials Science and Physics, China University of Mining and Technology, Xuzhou, 221116 People’s Republic of China

**Keywords:** Lithium-ion batteries, Silicon-based anodes, Prelithiation, Molecular dynamics simulations, Solvation structure

## Abstract

The solvation structure of Li^+^ in chemical prelithiation reagent plays a key role in improving the low initial Coulombic efficiency (ICE) and poor cycle performance of silicon-based materials. Nevertheless, the chemical prelithiation agent is difficult to dope active Li^+^ in silicon-based anodes because of their low working voltage and sluggish Li^+^ diffusion rate. By selecting the lithium–arene complex reagent with 4-methylbiphenyl as an anion ligand and 2-methyltetrahydrofuran as a solvent, the as-prepared micro-sized SiO/C anode can achieve an ICE of nearly 100%. Interestingly, the best prelithium efficiency does not correspond to the lowest redox half-potential (*E*_1/2_), and the prelithiation efficiency is determined by the specific influencing factors (*E*_1/2_, Li^+^ concentration, desolvation energy, and ion diffusion path). In addition, molecular dynamics simulations demonstrate that the ideal prelithiation efficiency can be achieved by choosing appropriate anion ligand and solvent to regulate the solvation structure of Li^+^. Furthermore, the positive effect of prelithiation on cycle performance has been verified by using an in-situ electrochemical dilatometry and solid electrolyte interphase film characterizations.

**Supplementary Information:**

The online version contains supplementary material available at 10.1007/s40820-023-01068-8.

## Highlights

- By selecting 4-methylbiphenyl as an anion ligand and 2-methyltetrahydrofuran as a solvent, the as-prepared micro-sized SiO/C anode can achieve an initial Coulombic efficiency of ~100%.
- Molecular dynamics simulations demonstrate that the ideal prelithiation efficiency can be achieved by choosing appropriate anion ligand and solvent to regulate the solvation structure of Li^+^.
- The positive effect of pre-lithiation on cycle performance has been verified by using an in-situ electrochemical dilatometry and solid electrolyte interphase film.

**Supplementary Information:**

The online version contains supplementary material available at 10.1007/s40820-023-01068-8.

## Graphical abstract

**Figure:**
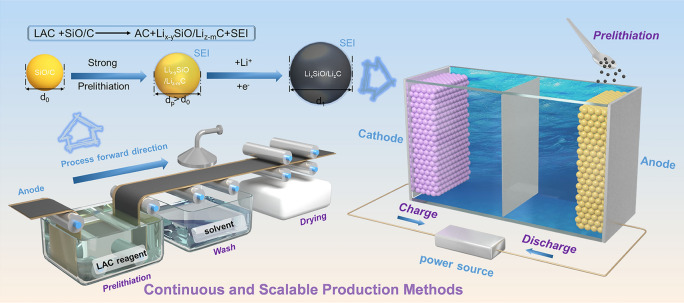

## Introduction

For realizing lithium-ion batteries (LIBs) with higher energy density, the silicon-based anode becomes a promising candidate in the market by virtue of its high theoretical specific capacity, low working voltage, and cost advantages [1–3]. However, the significant volume expansion of silicon-based material will destroy a three-dimensional (3D) conductive network of the electrode, resulting in the exfoliation of the active material from the current collector [4–6]. The formation of solid electrolyte interphase (SEI) film and the irreversible components will consume a large amount of active Li^+^ and suffer from a low initial Coulombic efficiency (ICE) [7–10]. Especially in the full battery, the limited Li^+^ will further deteriorate its electrochemical performance, which reduces energy density and cycle life. Besides, its low intrinsic electron conductivity (< 10^−5^ S cm^−1^) and sluggish Li^+^ diffusion rate (< 10^−14^ cm^2^ s^−1^) also lead to poor reaction kinetics [11–13].

Currently, sustained research efforts have been dedicated to optimizing and heightening its electrochemical property. Considering the production costs, complex synthetic procedures, and actual application scenarios, the designs of multi-level conductivity carbon skeletons for silicon-based materials would be an effective way to realize industrialization [5, 14–18]. However, a routine amorphous coating layer has a lower conductivity than crystalline carbon. At the same time, the amorphous carbon skeleton with plentiful defect sites will trap Li^+^ from participating in the subsequent cycles [19, 20]. In addition, the reserved buffer space significantly increases the specific surface area of the electrode, which would further consume a lot of electrolytes, resulting in a low ICE [19, 21–23]. Accordingly, it is particularly important to find an effective strategy to balance the relationship between cycle performance and ICE.

Premetallation is an effective strategy to improve ICE and enhance cycle stability of anodes [24–27]. Till now, various prelithiation methods have been developed to realize the commercial application [28–31]. It is a pity that these existing methods suffer from complicated processes, high cost, and compromising safety problems [32–34]. Fortunately, chemical prelithiation is a simple and safe method, which can achieve the prelithiation of silicon-based anodes at the electrode level through a redox reaction [35–37]. Nevertheless, the chemical prelithiation agent is difficult to dope active Li^+^ in silicon-based anodes due to their low working voltage and sluggish Li^+^ diffusion rate, so that the partial prelithiation is merely realized with the formation of SEI film [38–41].

To address those challenges, Zhang et al. reported an organolithium compound (Li-9, 9-dimethyl-9H-fluorene-tetrahydrofuran) with a low redox potential (0.18 V), enabling a high ICE of ~ 90.7% [40]. During the prelithiation process, some lithium ions can be embedded in active materials, which was accompanied by the SEI film formed. Lee et al. designed lithium–arene complex (LAC) reagents with low redox potential, according to the lowest unoccupied molecular orbital (LUMO) energy of aromatic compounds [39]. It is worth systematically exploring whether LAC reagents with a lower redox potential will achieve a stronger prelithiation effect of silicon-based anodes.

Based on the above considerations, the solvation structure of Li^+^ in LAC reagent was regulated by screening and regulating anion ligands and solvents, so as to achieve a desired prelithiation of silicon-based anodes. The relationships among E_1/2_, Li^+^ concentration and prelithiation efficiency were explored to guide the selection of anion ligands and solvents. Meantime, the effects of the Li^+^ solvation structure in LAC on prelithiation degree were investigated by theoretical calculation. Therefore, the ICE and cycle performance of the SiO/C anode were greatly improved by using a novel LAC reagent.

## Experimental Section

### Preparation of LAC Solutions

The LAC solution was prepared through dissolving lithium metal in as-prepared 1 M arene in tetrahydrofuran (THF, Macklin Inc., 99.5%) or 2-methyltetrahydrofuran (2-MTHF, Macklin Inc., 99%) solvent for 5 h at room temperature in an argon-filled glove box. It noted that Li^+^ and arene were uniformly mixed in a molar ratio of 4:1, which aims to supply an enough amount of Li sources for the formation of LACs. The arenes mainly include biphenyl (BP, TCI chemical, 99.5%), 4-methylbiphenyl (4BP, Macklin Inc., 98%), 4, 4′-dimethylbiphenyl (44'BP, Macklin Inc., 97%), and 2-methylbiphenyl (2BP, Macklin Inc., 99%). The above chemicals were used without further purification.

### Prelithiation of Anode

For the fabrication of electrodes, SiO/C (70 wt%), acetylene black (20 wt%), and polyacrylic acid (10 wt%) were dispersed in water to form a slurry, which was spread onto a Cu foil, following by drying in vacuum at 60 °C overnight. The oxygen-containing functional groups of polyacrylic acid can form hydrogen bonds with the hydroxyl groups on the surface of the silicon-based anodes, which is beneficial to improve the overall structural stability. The mass loading of the active material was 0.9–1.2 mg cm^−2^. During prelithiation process, the SiO/C anodes were immersed in the different kinds of LAC solutions for 10 min and then, rinsed with 1 M LiPF_6_ EC/DEC (1:1 *v*/*v*) electrolyte with 5 vol% FEC to quench reaction between LACs and anodes. The molar ratio of LAC: SiO was fixed to 6: 1. Finally, the prelithiated SiO/C (p-SiO/C) anode was obtained. The corresponding prelithiated anode can be obtained by replacing the anode material with micron-sized SiO, nano-sized Si (nSiO), and nano-sized Si (nSi) particles.

### Material Characterization

The chemical composition and bonding environment of the material were analyzed by X-ray diffraction (XRD, PANalytic B. V., Cu Kα), and X-ray photoelectron spectroscopy (XPS, Perkin Elmer PHI 550) to determine the crystal structure. The morphologies of samples and electrodes before and after cycling were characterized by the scanning electron microscope (SEM, HITACHI S-4800). The transmission electron microscope (TEM, FEI Tecnai-20) and the energy dispersive spectroscopy were further used to investigate the samples and the electrodes, respectively. The Li^+^ concentration of LAC solution was measured by inductively coupled plasma optical emission spectrometer (ICP-OES, PerkinElmer ICP 2100).

## Results and Discussion

### Characterization and Prelithiation of SiO/C Anodes

**Fig. 1:**
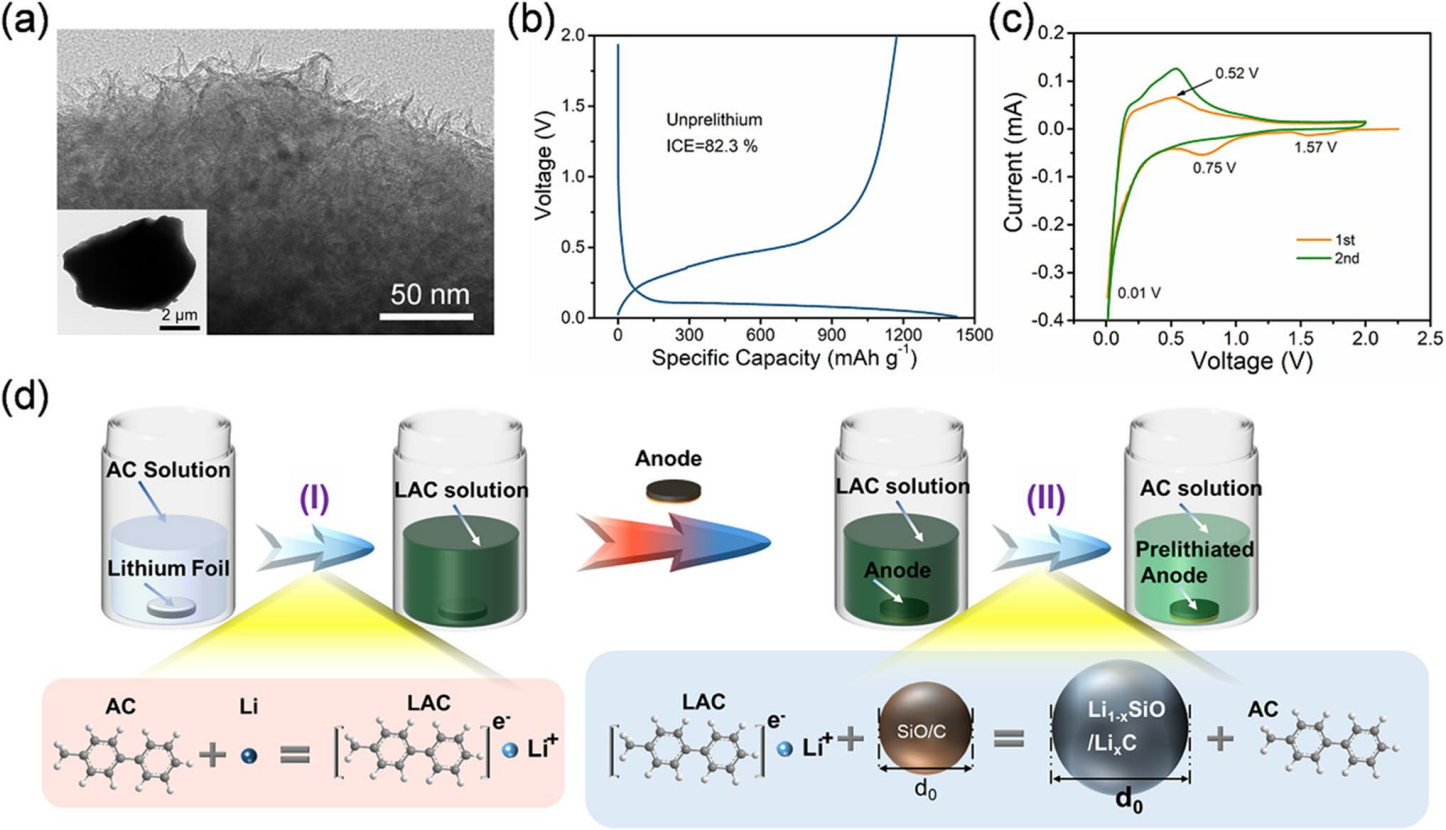
**a** TEM images of SiO/C particle. **b** Initial discharge–charge profile and **c** CV curve of SiO/C anodes. **d** Schematic illustration of the prelithiation process of SiO@C electrode

Considering that micron-sized SiO particle shows a higher energy density and low cost, we chose commercial micron-sized SiO/C materials as anodes. In Fig. 1a, the irregular SiO particles were tightly wrapped by a graphene-like carbon layer, and their sizes were distributed between 5 and 10 μm (Fig. S1a, b). The XRD test was executed to determine the component of SiO/C material. As is shown in Fig. S1c, the strong diffraction peaks of SiO/C material at 28.4° and 47.3° correspond to the (111) and (220) crystal planes of crystalline silicon (JPCDS No. 01-077-2109) [20, 42]. The wide diffraction peaks between 20° and 26° belong to the graphene-like carbon layer. In addition, other impurities were not found, except the diffraction peaks of crystal SiO_2_. Afterward, the electrochemical performances of SiO/C anodes were evaluated. Figure 1b shows that the initial discharge and charge specific capacities of pristine SiO/C anode are 1,424.7 and 1,172.6 mAh g^−1^ at 0.1 A g^−1^, and the corresponding ICE is 82.3%. The unsatisfactory ICE is attributed to irreversible peaks, which corresponds electrolyte decomposition at 1.57 V and the formation of irreversible lithium silicate and lithium oxide at 0.75 V (Fig. 1c). The cathodic peaks 0.01 V and the anodic peak at 0.52 V are associated with the conversion of the Li–Si phase, respectively.

To solve this issue, prelithiation is an effective method to compensate the loss of active Li^+^. The prelithiation process of SiO/C material is mainly divided into two steps: (I) Preparation of LAC reagent and (II) the lithiation of SiO/C anode, which is systematically explored to guide the design of LAC reagents. As is depicted in Fig. 1d, the lithium metal can react with 1 M arene complex (AC) solution to form LAC (AC + Li → LAC), along with the solution color changing from transparent to dark green in step (I) [43–45]. When the electrode was immersed in the above solution, Li^+^ and electrons transferred from LAC to the SiO/C anode in step (II), corresponding to the following reaction: LAC + SiO/C → AC + Li_1−x_SiO@Li_x_C. It may be reasonably expected that AC should have a strong ability to obtain electrons to ensure the reaction with lithium metals and also have a strong ability to release electrons to ensure that Li^+^ can be released smoothly. Meanwhile, the prelithiation process of anode is jointly controlled by the prelithiation ability of LAC reagent and the Li^+^ diffusion path of active material.

**Fig. 2:**
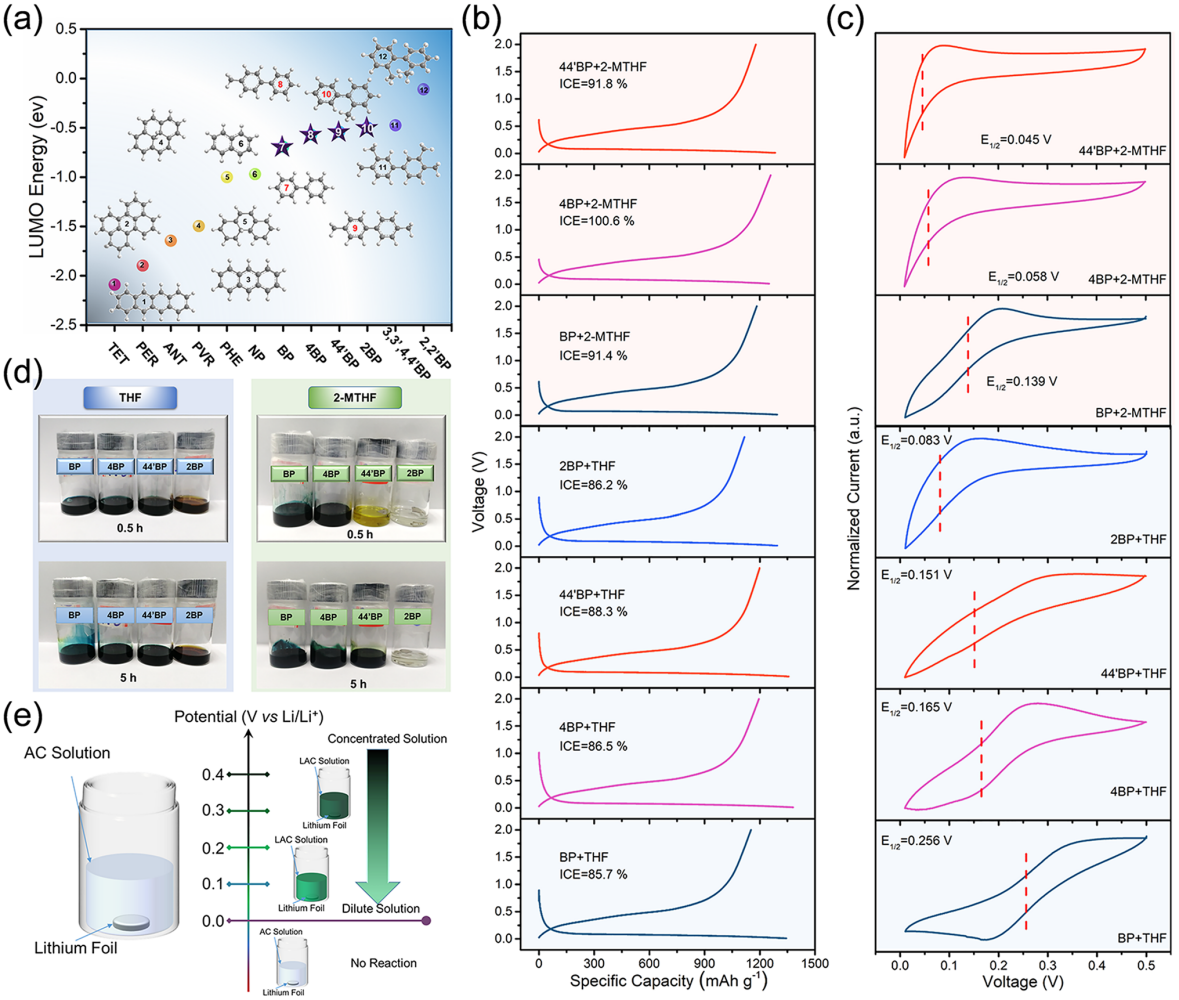
**a** LUMO energies of different arenes. **b** Initial discharge–charge profiles of p-SiO/C anodes immersed in different prelithiation reagents. **c** CV curves of Cu electrode in different 0.1 M LAC reagents containing 1 M LiPF_6_ at 20 mV s^−1^. **d** Digital photographs of the color change of LAC reagents. **e** Schematic diagram of the reaction possibility between lithium metal and AC solution

According to previous researches, the electron cloud density of alkyl is larger than that of hydrogen, and the electrons of the carbon–hydrogen bonds in the alkyl group are easily conjugated to the adjacent *π*-electron system, and the electron delocalization occurs [39, 46, 47]. Therefore, the LUMO energy of arene is crucial to fabricating the LAC reagent with a low *E*_1/2_. Herein, we have selected different arenes to optimize the *E*_1/2_ value for the best prelithiation efficiency according to LUMO energies (Fig. 2a). The LAC reagents were prepared through dissolving lithium metal in as-prepared 1 M AC solutions for 5 h. The selected arenes mainly include BP, 4BP, 44'BP, and 2BP. And the solvents are THF and 2-MTHF. After being immersed in different LAC reagents for 10 min, the ICEs of SiO/C anodes were significantly improved (Fig. 2b). Among them, the prelithiation reagent (4BP + 2-MTHF) prepared with 4BP as an anion ligand and 2-MTHF as a solvent shows the best prelithiation efficiency, and the corresponding ICE of p-SiO/C is the highest (100.6%). The ICEs of SiO/C anodes prepared by other prelithiation reagents were 85.7% (BP + THF), 91.4% (BP + 2-MTHF), 86.5% (4BP + THF), 88.3% (44′BP + THF), 91.8% (44′BP + 2-MTHF), and 86.2% (2BP + THF), respectively. Because the prelithiation efficiency of SiO/C anode is related to the *E*_1/2_ of LAC reagent, CV tests were performed in Fig. 2c. Interestingly, the *E*_1/2_ of LAC reagent (4BP + 2-MTHF) is 0.058 V and not the lowest, while the prelithiation efficiency of SiO/C anode is the best.

To better analyze the experimental results, the color changes observed in the preparation process of LAC reagents are shown in Fig. 2d. When THF was used as a solvent, the LAC reagents with BP, 4BP, and 44’BP as anion ligands show darker color after 5 h, but the LAC reagent with 2BP as anion ligand shows a lighter color (brown). This phenomenon is more obvious when 2-MTHF was used as solvent. After 0.5 h, the LAC reagents prepared with BP and 4BP as anionic ligands show dark colors. The LAC reagent prepared with 44'BP was light yellow. And the LAC reagent configured with 2BP as anionic ligand was transparent. Even after 5 h, the color of LAC reagent with 2BP still remains unchanged. In order to prove that the color change was not directly related to the solvent, lithium metal was soaked in pure THF or 2-MTHF for 24 h. It can be seen from Fig. S2 that there is no redox reaction between lithium metal and pure THF/2-MTHF solvents. Because *E*_1/2_ is less than or close to 0 V, no reaction occurs between lithium metal and AC solution [39]. Based on the above phenomena, we have deduced the following rules in Fig. 2e. As the *E*_1/2_ of LAC reagents decreases, the reaction possibility between lithium metal and AC solution becomes smaller, and the concentration of Li^+^ decreases.

### Exploration of Prelithium Mechanism

**Fig. 3:**
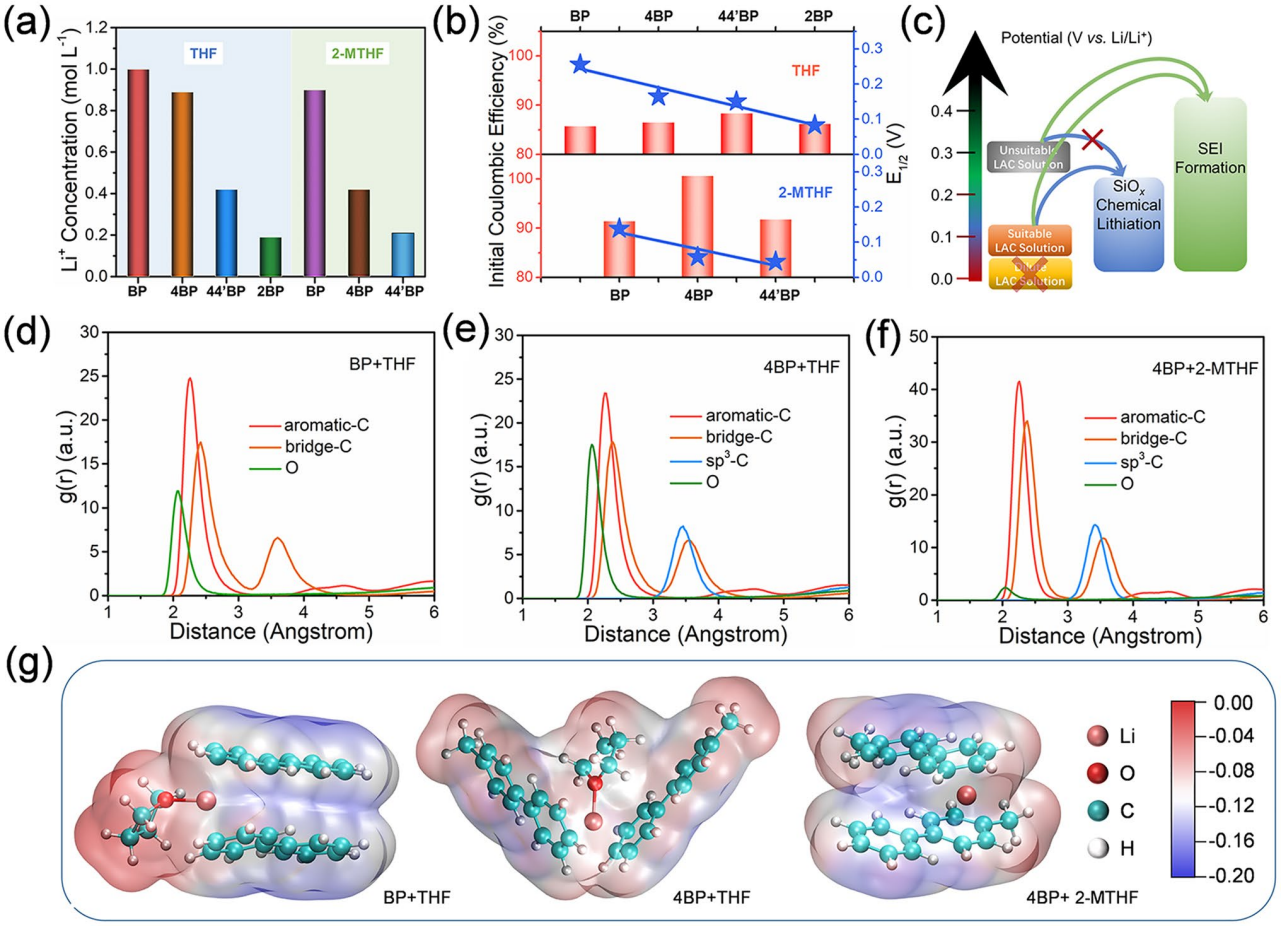
**a** Li^+^ concentrations of LAC reagents. **b** ICE and *E*_1/2_ of p-SiO/C anodes. **c** Schematic diagram of the relationship between LAC reagent and prelithiation efficiency of SiO/C anode. **d, f** Calculated radial distribution functions and **g** the corresponding first solvation sheath structures with electrostatic potential mappings of different LAC reagents

Subsequently, ICP-OES tests were performed to detect the Li^+^ concentration of the LAC reagents (Fig. 3a). When THF or 2-MTHF was used as a solvent, the LAC reagents prepared with BP, 4BP, and 44'BP anionic ligands show a decreasing trend of Li^+^ concentration, which was positively associated with *E*_1/2_ of the p-SiO/C anode (Fig. 3b). When THF is used as the solvent, *E*_1/2_ and Li^+^ concentration decreased with the increase in LUMO energy, but the ICEs of SiO/C anodes first increased and then decreased. This trend is more pronounced when 2-MTHF is used as the solvent. Therefore, the above results confirm the existence of optimal value (*E*_1/2_) for the best prelithiation efficiency. As illustrated in Fig. 3c, the prelithiation efficiency of SiO/C anode first increased and then decreased, with the decrease in *E*_1/2_ of LAC reagents. Until the *E*_1/2_ of LAC reagent is close to 0.05 V, the SiO/C anode can realize the strongest prelithiation efficiency. Provided that the *E*_1/2_ of LAC reagent continues to decrease, the Li^+^ concentration will decrease sharply. As a result, the SiO/C anode is difficult to get enough active Li^+^ to achieve a desired prelithiation.

However, when 4BP was used as anion ligand, there are still doubts. Compared with THF as a solvent, LAC reagent with 2-MTHF as a solvent exhibits a lower Li^+^ concentration, but the ICE of the SiO/C anode is significantly enhanced. Therefore, the desolvation kinetics of Li^+^ in LAC reagent was discussed [10, 41]. The SiO/C particles were surrounded by Li^+^-solvent. With the removal of solvent and anion ligands, Li^+^ was embedded into SiO/C particles to achieve prelithiation (Fig. S3). Based on the ab initio molecular dynamics simulations, the solvation structures of Li^+^ in LACs were optimized to obtain their first solvation sheath structure. The initial structures of LACs were set up by randomly placing anion/solvent molecules based on their molar ratios. The radial distribution functions (RDFs) of LACs usually refer to the distribution probability of other granules in space given the coordinates of a granule. In Fig. 3d–f, it should be noted that the aromatic-C, bridge-C, and *sp*^3^-C are used to locate the position of BP or 4BP, and O is used to locate the position of THF or 2-MTHF. Combined with the corresponding integrals in Fig. S4, each Li^+^ is coordinated with two benzene rings on average, and BP + THF system is slightly larger than 4BP + THF system. Coordinated with one O atom, 4BP + THF system is slightly larger than BP + THF system. However, each Li^+^ in 4BP + 2-MTHF system is coordinated with about two benzene ring planes on average, and the O atom is almost not involved in the coordination. According to the above results, the first solvation sheath structures with electrostatic potential mappings of the three systems are shown in Fig. 3g. Based on quantum chemical calculations, the 4BP + 2-MTHF system exhibits the lowest desolvation energies of Li^+^ (604.58 kJ mol^−1^). By comparison, the desolvation energies of Li^+^ in other two systems are 649.24 kJ mol^−1^ and 655.97 kJ mol^−1^, respectively. And compared to the other two systems, the 4BP + 2-MTHF system displays a more uniform electrostatic potential field. The optimized electron density distribution is favor to reduce the binding force of Li^+^ and anionic ligands, which reduce *E*_1/2_ values [39]. Therefore, the ideal prelithiation efficiency can be achieved by choosing appropriate anion ligands and solvents to regulate the solvation structure, which can balance the relationship among *E*_1/2_, Li^+^ concentration and desolvation energy.

**Fig. 4:**
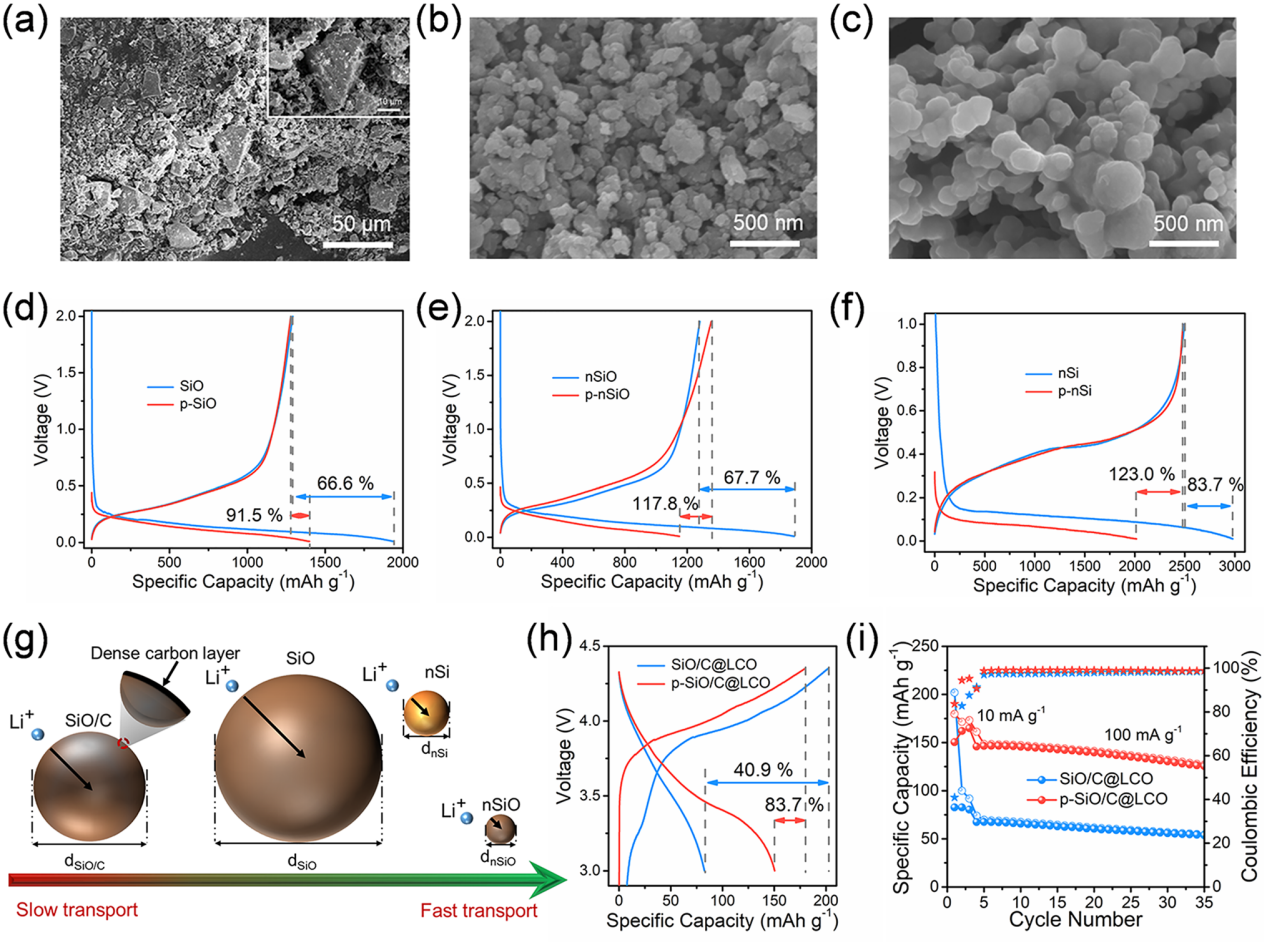
SEM images of **a** SiO, **b** nSiO, and **c** nSi particles. Initial discharge–charge profiles of **d** SiO and p-SiO anodes, **e** nSiO and p-nSiO anodes, and **f** nSi and p-Si anodes. **g** Influencing factors of prelithiation efficiency of SiO/C, SiO, nSiO, and nSi anodes. **h** Initial charge–discharge profiles and **i** cycle performances of SiO/C@LCO and p-SiO/C@LCO full cells

Except for the prelithiation ability of LAC reagent, different silicon-based materials were tried to verify the roles of the ion diffusion path inside active materials in the prelithiation efficiency. The morphologies of micron-sized SiO, nSiO, and nSi particles were observed by SEM test. In Fig. 4a, commercial SiO particles show a non-uniform size, and their distribution varies from a dozen microns to dozens of microns. To compare the effect of particle sizes on the degree of prelithiation, micron-sized SiO was broken into nSiO particles by high-energy ball milling (HEBM, Fig. 4b). Figure 4c shows nSi particles with the same nanoscale. Subsequently, SiO, nSiO, and nSi anodes were also immersed in LAC (4BP + 2-MTHF) reagent for 10 min. Figure 4d displays that the ICEs of SiO and p-SiO anodes are 66.6% and 91.5%, respectively. With the size of commercial SiO particle reaching nanometer level, the prelithiation efficiency of nSiO is greatly improved (Fig. 4e), and the ICE is increased from 67.7% (nSiO) to 117.8% (p-nSiO). After nanorization, the ICE of nSiO increases to 40.1%, which is much higher than that of SiO (24.9%). By contrast, the ICE of prelithiated nSi anode material is as high as 123.0%, and the corresponding ICE increase is 39.3% (Fig. 4f). According to the above results, we have assumed that the prelithiation efficiency of electrode materials is also governed by the Li^+^ diffusion path (Fig. 4g). With the decrease in particle size, it takes less time for Li^+^ to diffuse from the particle surface to the material interior [48, 49]. Meanwhile, a larger contact area with the prelithiation solvent is also conducive to improving the prelithiation efficiency. And the dense carbon layer coated on the surface of SiO particles will hinder the rapid embedding of Li^+^, thereby weakening the prelithiation efficiency. If we can combine the modification process with the prelithiation technology, the prelithiation time of silicon-based anodes will be shortened to reduce costs.

To prove the significance of prelithiation in the realization of high-energy density, we have assembled full cells by using the SiO/C and p-SiO/C as anodes and the commercial LiCoO_2_ (LCO) as a cathode. In Fig. S5a, the initial charge–discharge capacities of LCO cathode were 178.3 and 167.1 mAh g^−1^, respectively. After 35 cycles, the reversible specific capacity of the LCO cathode decreased slightly, which was stable at 171.8 mAh g^−1^ (Fig. S5b). After capacity and voltage matching, the voltage range of the full cells is 3–4.35 V. The full cells were first activated at 10 mA g^−1^. As is shown in Fig. 4h, the p-SiO/C@LCO full cell exhibits initial charge–discharge capacities of 179.8 and 150.4 mAh g^−1^, corresponding to a high ICE of 83.7%. Due to the large amount of active Li^+^ loss caused by the initial irreversible process, the ICE of SiO/C@LCO full cell is only 40.9%. After 35 cycles in Fig. 4i, the reversible capacity of p-SiO/C@LCO full cell (125.4 mAh g^−1^) is much higher than that of SiO/C@LCO (53.7 mAh g^−1^).

### Effect of Prelithiation on Cycle Stability

**Fig. 5:**
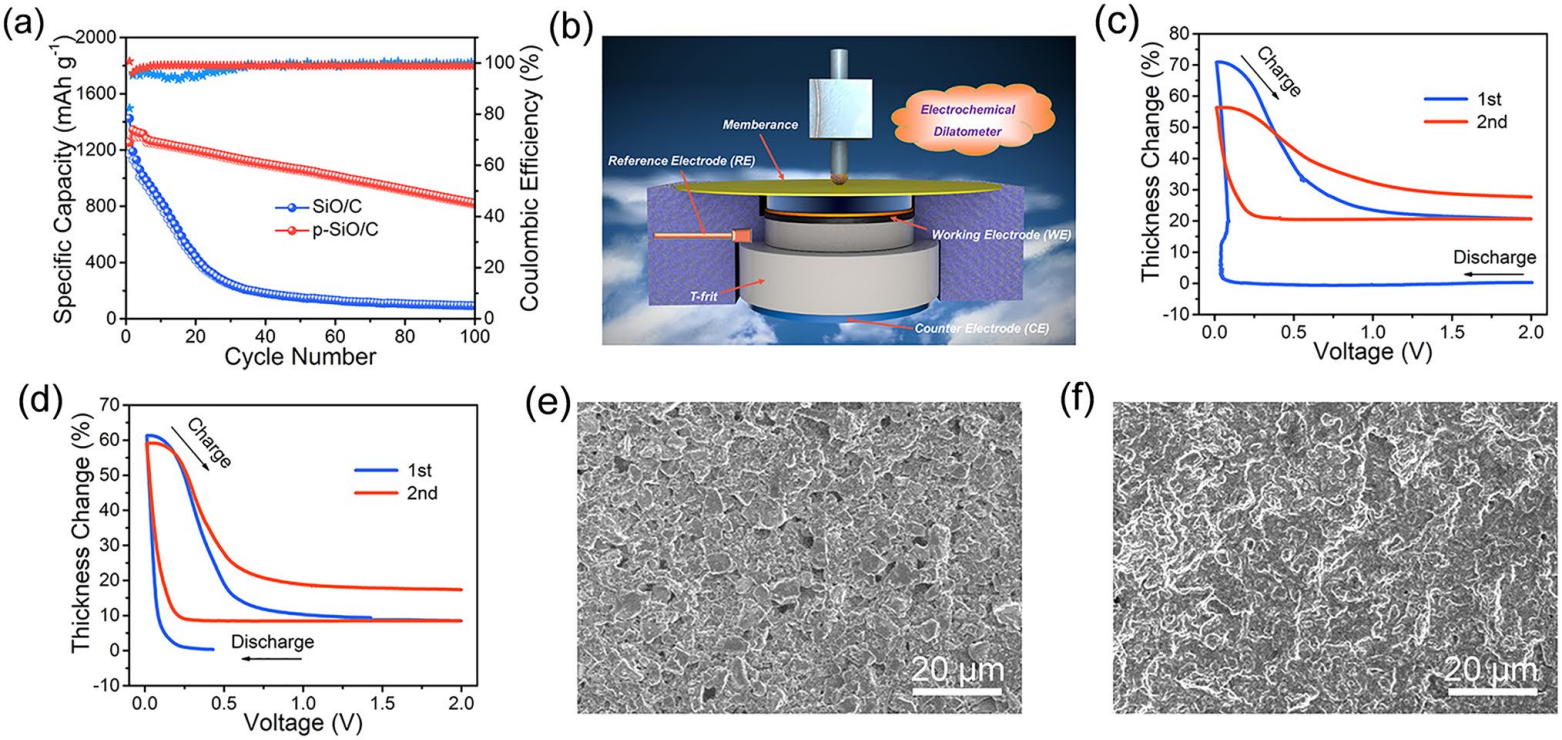
**a** Cycle performances of SiO/C and p-SiO/C anode. **b** Schematic diagram of the in-situ electrochemical dilatometry. **c** Voltage-thickness curves of SiO/C and **d** p-SiO/C anodes. SEM images of **e** SiO/C and **f** p-SiO/C anodes

Considering the good stability of the LCO cathode, the difference in capacity retention of full cell is attributed to the prelithiation of SiO/C particles. Figure 5a shows the cycle performances of SiO/C and p-SiO/C anodes at 0.2 A g^−1^. After 100 cycles, the reversible capacity of p-SiO/C anode is 813.9 mAh g^−1^ and the corresponding capacity retention is 64.6%, whereas the capacity retention of SiO/C anode is only 7.9%. The in-situ electrochemical dilatometer was used to monitor the thickness changes of SiO/C and p-SiO/C anodes during cycling (Fig. 5b) [50, 51]. The voltage-thickness curves of SiO/C and p-SiO/C anodes were shown in Fig. 5c, d. During the first cycle, the thickness variation of SiO/C anode is 70.8%, which is higher than that of p-SiO/C anode (61.5%). These results demonstrate that the p-SiO/C anode successfully realizes Li^+^ pre-embedding, so the first thickness change decreases. In addition, during the first two cycles, p-SiO/C anode shows highly reversible thickness variations, which are 52.9% and 41.2%, respectively. By comparison, the reversible thickness variations of SiO/C anode were only 49.9% and 28.7%. The main reason is that the prelithiation of SiO/C anode can prevent effectively mechanical degradation caused by repeated volume changes. At the same time, the pre-generation of SEI film can also reduce the loss of Li^+^ by the irreversible reaction. In order to verify our view, we conducted SEM tests on SiO/C and p-SiO/C anodes. In Fig. 5e, different shapes of SiO/C particles are still angular with abundant pore structures on the electrode surface. After prelithiation, the edges of SiO/C particles disappear owing to pre-expansion, and the surface of particles becomes smooth. Besides, the pore structure on the surface of p-SiO/C electrode decreased (Fig. 5f). These phenomena further confirm the positive effect of prelithiation on the improvement of cycle stability.

**Fig. 6:**
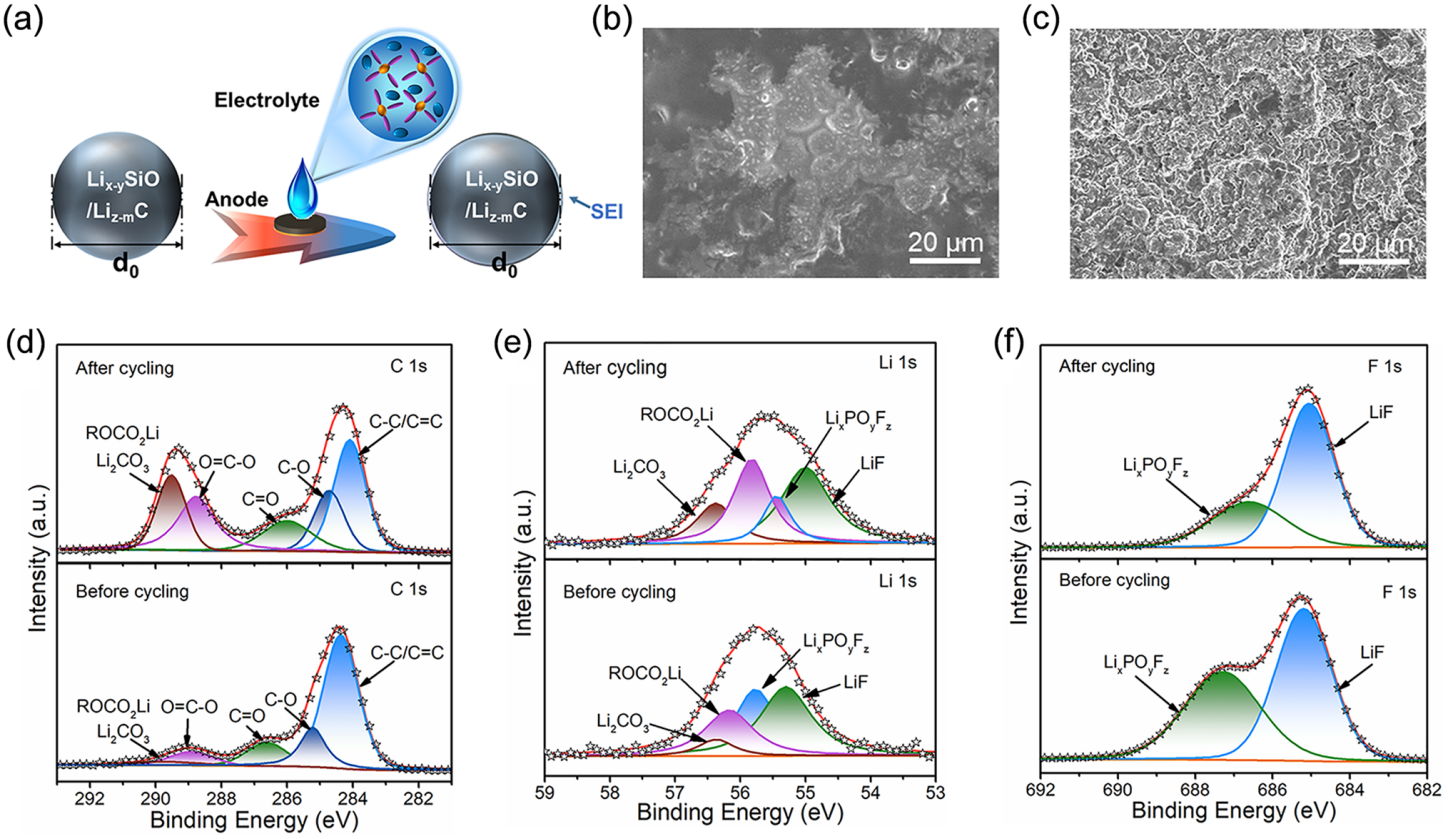
**a** SEI film constructed by contacting the prelithiated electrode with the electrolyte. SEM images of p-SiO/C anodes **b** before cycling and **c** after cycling. Refined XPS high-resolution spectra of **d** C 1*s*, **e** Li 1*s* and **f** F 1*s* of p-SiO/C anodes before and after cycling

In addition. the SEI film on electrode surface plays an important role in the optimization of electrochemical performance. In general, the prelithiated electrode needs to be in contact with commercial electrolyte to form a SEI film in advance, so as to reduce the active Li^+^ loss in full cells (Fig. 6a). Nevertheless, the cycle performance of p-SiO/C anode is still not ideal. Therefore, ex-situ characterizations were carried out to reveal the failure mechanism of p-SiO/C anodes before and after cycling. Firstly, the morphologies and structures of p-SiO/C anodes were characterized by SEM tests. In Fig. 6b, the surface of p-SiO/C electrode is smooth and flat before cycling, and the surface morphology of p-SiO/C material can be seen probably. This is due to the formation of a thin SEI film on the surface of the prelithiated electrode after contacting with the electrolyte. After multiple cycles (Fig. 6c), the repeated volume expansion of p-SiO/C particles results in particle displacement and SEI fragmentation. Therefore, the surface of p-SiO/C electrode becomes uneven, indicating there is a thick SEI film. TEM tests were also further used to reveal the SEI film on the surface of p-SiO/C particles. Before and after cycles, p-SiO/C particles still maintained the integrity of the structure (Fig. S7a, b). Figure S7c shows that p-SiO/C particles were covered by a thin amorphous SEI film before cycling. And after multiple cycles, the SEI film became thicker and uneven (Fig. S7d). The corresponding STEM image and elemental mappings of p-SiO/C anodes after cycles were illustrated in Fig. S8, where the uneven distribution of fluorine further confirms the unevenness of the SEI film. The above phenomenon is consistent with SEM results.

Subsequently, the SEI components of p-SiO/C anodes before and after cycling were analyzed. XPS test was also used to further reveal the composition difference between the SEI film formed by prelithiation and the SEI film formed after crushing. Figure 6d-f are the C 1*s*, Li 1*s*, and F 1*s* spectra of p-SiO/C anodes before and after cycling [10, 52–54]. Compared with that of p-SiO/C anode before cycling, the peak intensities of ROCO_2_Li and Li_2_CO_3_ after cycling were significantly enhanced, indicating that a large number of EC and DEC decomposed during cycling and participated in the formation of SEI film (Fig. 6d). The above results are further verified in Li 1*s* spectrum (Fig. 6e). LiF mainly comes from the decomposition of FEC and LiPF_6_, and Li_x_PO_y_F_z_ mainly comes from the decomposition of LiPF_6_ [55]. Herein, the high electrical insulation and high mechanical strength of LiF can avoid electrolyte decomposition and adapt to repeated volume changes of Si anodes. At the same time, the formation of nanoscale heterojunctions among LiF and other inorganic components can meet the needs of SEI film for ionic conductivity, even though LiF is an ionic insulator. Therefore, the improved SEI film exhibits good cycle stability and fast ion permeability [10, 56]. In Fig. 6e, f, the Li_x_PO_y_F_z_ peak area of p-SiO/C anode before cycling accounts for more than that of p-SiO/C anode after cycling, while the LiF peak area accounted for less. The above results indicate that it is difficult for the SEI film generated in the prelithiation process to obtain sufficient mechanical toughness to improve the volume expansion of the silicon-based anode. Therefore, the reasonable construction of SEI component in the prelithiation process is also very important for the optimization of cycle performance.

## Conclusions

In summary, the desirable prelithiation of the silicon-based anode can be achieved by selecting different anion ligands and solvents to regulate solvation structure of Li^+^ in LAC reagent. The prelithiation process is jointly controlled by the prelithiation ability of LAC reagent and the lithiation process of active material. The relationships among *E*_1/2_, Li^+^ concentration and prelithiation efficiency were explored for the sake of guiding the selection of anion ligands and solvents. Meanwhile, theoretical calculation demonstrates that the stronger Li^+^ desolvation ability leads to better prelithiation effect when the *E*_1/2_ is low enough. Therefore, the ICE of commercial SiO/C anode increased from 82.3% to 100.6% by using a suitable LAC reagent. Moreover, the ICE of nSiO anode with the shortest ion diffusion path increased from 67.7% to 117.8%, demonstrating that the prelithiation efficiency is related to the Li^+^ diffusion path. Furthermore, the in-situ electrochemical dilatometry reveals that prelithiation improves the cycle stability of SiO/C anode. Through the analysis of SEI morphology evolution and composition, the shortcomings of current chemical prelithiation technology are further revealed. Consequently, the combined chemical prelithiation with artificial SEI construction can provide an effective tool to improve the ICE and the cycle performance of silicon-based anodes.

### Supplementary Information

Below is the link to the electronic supplementary material.

Supplementary file1 (PDF 606 KB)
