# Supplementary material for: Regulating the Solvation Structure of Li+ Enables Chemical Prelithiation of Silicon-Based Anodes Toward High-Energy Lithium-Ion Batteries
Source: Nanomicro Lett. 2023 Apr 18;15:107. doi: 10.1007/s40820-023-01068-8 (PMC10113421; doi:10.1007/s40820-023-01068-8)
Supplement: Supplementary file 1 — Supplementary file1 (PDF 606 KB) [file 40820_2023_1068_MOESM1_ESM.pdf]

Supporting Information for

Regulating the Solvation Structure of Li^+ Enables Chemical Prelithiation of Silicon-Based Anodes Toward High-Energy Lithium-Ion Batteries

Wenjie He[1, 2], Hai Xu[1], Zhijie Chen[1], Jiang Long[1], jing Zhang[1], Jiangmin Jiang[1, 3, *], Hui Dou[1, *], Xiaogang Zhang[1, *]

[1]Jiangsu Key Laboratory of Electrochemical Energy Storage Technologies, College of Material Science and Technology, Nanjing University of Aeronautics and Astronautics, Nanjing 210016, P. R. China

[2]School of Materials Science and Engineering, Henan Polytechnic University, Jiaozuo 454003, P. R. China

[3]Jiangsu Province Engineering Laboratory of High Efficient Energy Storage Technology and Equipments, School of Materials Science and Physics, China University of Mining and Technology, Xuzhou 221116, P. R. China

*Corresponding authors. E-mail: azhangxg@163.com (Xiaogang Zhang); dh_msc@nuaa.edu.cn (Hui Dou); jiangmin326@163.com (Jiangmin Jiang)

S1 Experimental Section

S1.1 Half-Cell Assembly and Electrochemical Measurements

The electrochemical performances were characterized by assembling CR2032 coin cells with lithium foil as the counter electrode and (prelithiated) anode as working electrode. 1M $LiPF_6$ EC/DEC (1:1 v/v) electrolyte with 5 vol% FEC was used as the electrolyte. Galvanostatic charge/discharge measurements were performed in a potential range of 0.01-2.0 V using a multichannel battery testing system (LAND CT2001A). Cyclic voltammetry (CV) and electrochemical impedance spectroscopy (EIS) measurements were tested with electrochemical workstation system (Biologic Science Instruments, Claix, France).

S1.2 Full-Cell Assembly and Electrochemical Measurements

SiO/C and p-SiO/C were used as anodes and high voltage $LiCoO_2$ (LCO) were used as cathode. The mass ratio of cathode to anode was around 3:1, and the weight ratio of LCO: carbon black: PVDF was 8:1:1 in the cathode. The full-cells were galvanostatic charged/discharged at 0.1 A g^{-1} in the electrochemical window of 3-4.35 V.

S1.3 Test of LAC redox potential

The electrolyte used in CV tests was 1 M $LiPF_6$ in THF/2-MTHF solvent containing 0.1 M Li-arene. The Cu foil and lithium metal were used as the counter electrode and as working electrode, respectively. CV curves were recorded at a scanning rate of 20 mV s^{-1} at room temperature.

S1.4 Computational Details

Molecular dynamics (MD) simulations are performed with Gromacs, version 2019.6 (*SoftwareX 1, 19– 25, (2015)*). Quantum chemical calculations (including desolvation energy and electrostatic potential mapping), are carried out with Gaussian 16, Multiwfn (*J. Comput. Chem., 33, 580 (2012)*), and VMD program (*J. Mol. Graph 14, 33–38 (1996)*). The MD simulation systems consist of 100 lithium cations, 100 BP/MBP anions and 300 THF/MTHF solvent molecules. All species during MD simulations are modelled by GAFF force field (*J. Comput. Chem. 25, 1157–1174, (2004)*), with the RESP atomic chargs derived by Multiwfn program by fitting to electrostatic potential calculated at the B3LYP-D3/6-31+G* level of theory. Lennard-Jones (LJ) potential truncated at 1.2 nm is used to calculate van der Waals interactions. LJ parameters between unlike atoms are derived by Lorentz–Berthelot mixing rules. Coulombic interactions are calculated with the particle-mesh Ewald method. Initial conformations are energy minimized by steepest descent algorithm, followed by 20-ns production runs at 298.15 K and 1 bar, with v-rescale thermostat and Berendsen barostat. The time step is 1 fs. Three dimensional periodic boundary conditions are considered during simulations.

The average solvation structure around a lithium cation is determined by the integrated radial distribution function analysis (coordination number) of a MD trajectory. On that basis, the solvation strucutures are extracted from the three MD simulations. Subsequently, the structures are optimized at the B3LYP-D3/6-31+G* level of theory, and further calculated at the B3LYP-D3/6-311G** level of theory. The electrostatic potential as well as the molecular isosurface are analyzed by Multiwfn and visualized by VMD program.

S2 Supplementary Figures

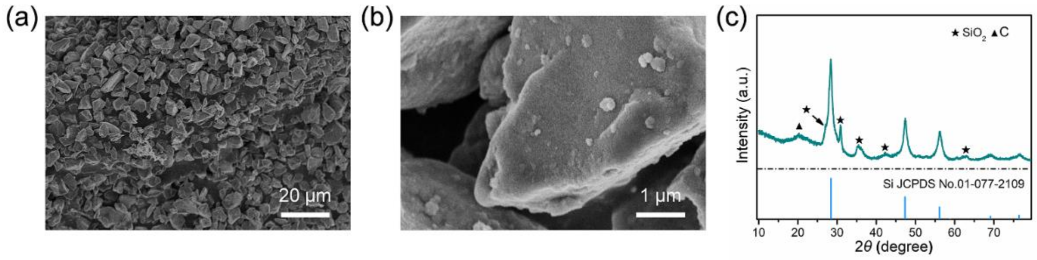

Fig. S1 (**a-b**) SEM images and (**c**) XRD pattern of the SiO/C particles

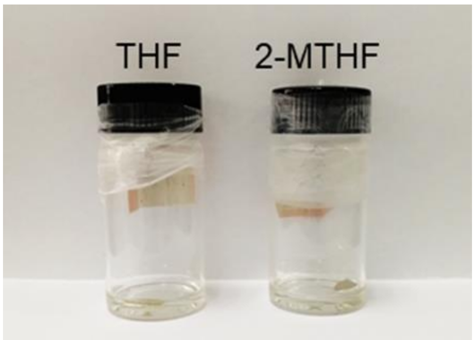

Fig. S2 Digital photograph of lithium metals immersed in THF or 2-MTHF solvents for 24 h

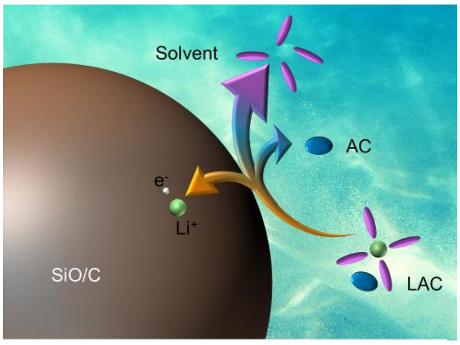

Fig. S3 The schematic diagram of Li^+ desolvation process in LAC reagents

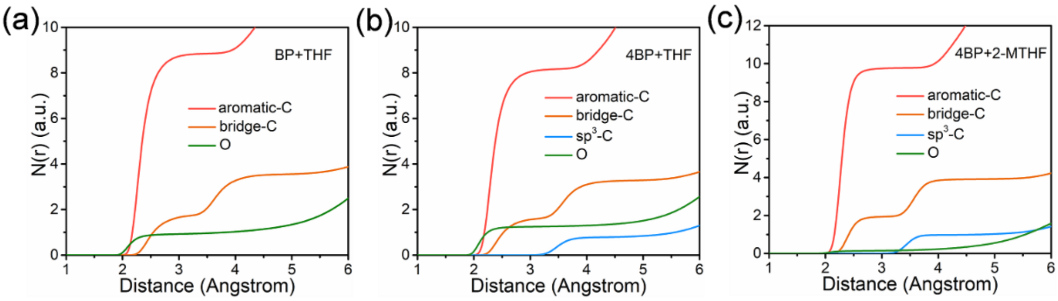

Fig. S4 The corresponding integrals of calculated radial distribution functions

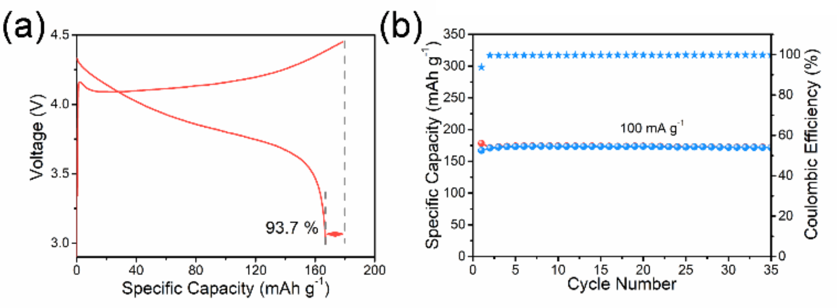

Fig. S5 (**a**) Initial discharge-charge profiles and (**b**) the cycling performance of LCO

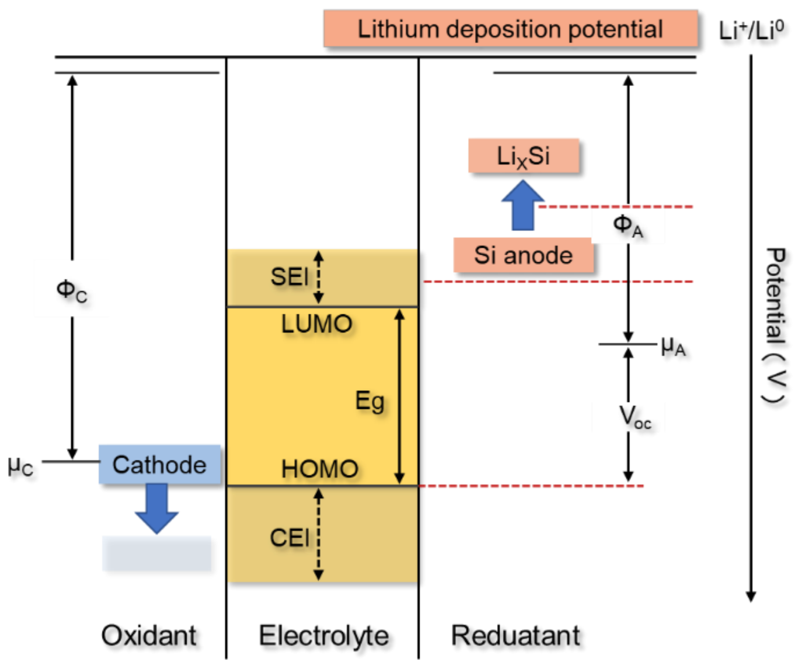

Fig. S6 The thermodynamic stability of the solid-liquid interface

The construction of SEI film during the prelithiation process can compensate for Li^+ loss and solve the solid-liquid interface problem, which is an effective means to achieve stable cycle and high capacity retention. As shown in **Fig. S6**, the thermodynamic stability of the solid-liquid interface depends on the energy difference between the Fermi level of anode and the electrolyte LUMO level. When the LUMO energy level of the electrolyte is lower than the electrochemical potential (μ_A) of the negative electrode, the electrolyte will spontaneously reduce and consume Li^+, resulting in the passivation of the electrode surface to form a SEI film.

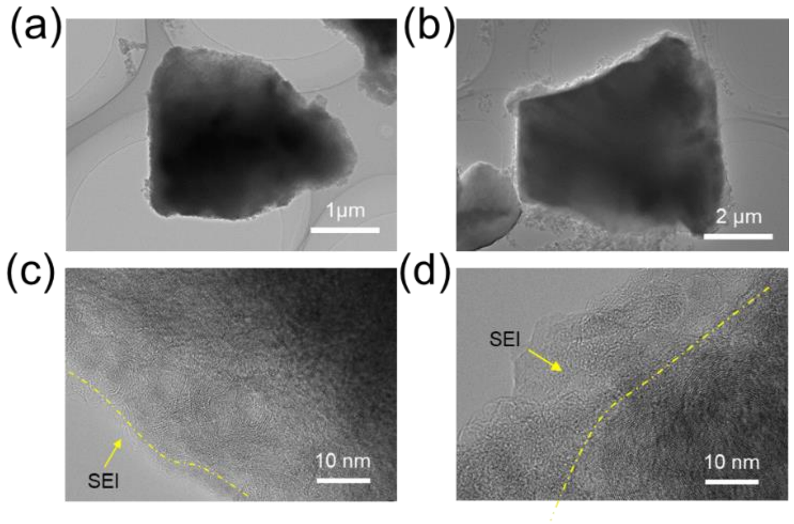

Fig. S7 TEM images of p-SiO/C anodes (**a, c**) before and (**b, d**) after 100 cycles

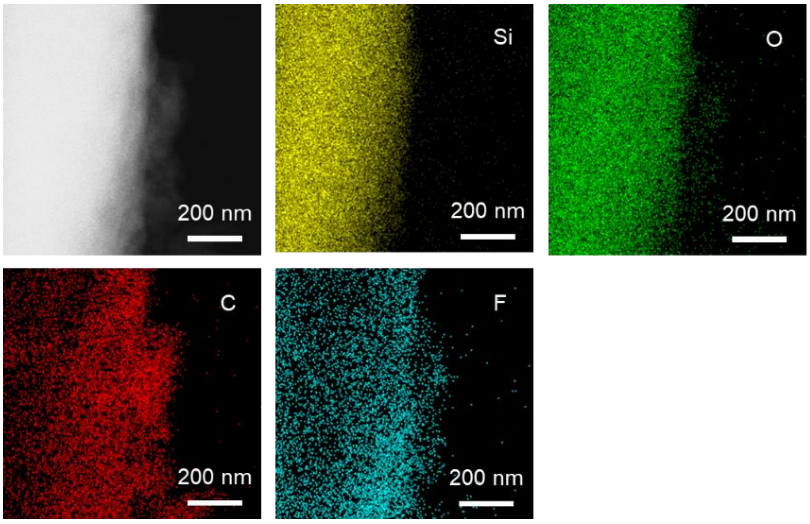

Fig. S8 The corresponding STEM image and elemental mappings of p-SiO/C anodes after 100 cycles
